# Diverse and abundant multi-drug resistant *E. coli* in Matang mangrove estuaries, Malaysia

**DOI:** 10.3389/fmicb.2015.00977

**Published:** 2015-09-29

**Authors:** Aziz Ghaderpour, Wing Sze Ho, Li-Lee Chew, Chui Wei Bong, Ving Ching Chong, Kwai-Lin Thong, Lay Ching Chai

**Affiliations:** ^1^Faculty of Science, Institute of Biological Science, University of MalayaKuala Lumpur, Malaysia; ^2^Institute of Ocean and Earth Sciences, University of MalayaKuala Lumpur, Malaysia

**Keywords:** *E. coli*, Matang mangrove estuaries, antibiotic resistance, phylogenetic groups

## Abstract

*E.*coli, an important vector distributing antimicrobial resistance in the environment, was found to be multi-drug resistant, abundant, and genetically diverse in the Matang mangrove estuaries, Malaysia. One-third (34%) of the estuarine *E. coli* was multi-drug resistant. The highest antibiotic resistance prevalence was observed for aminoglycosides (83%) and beta-lactams (37%). Phylogenetic groups A and B1, being the most predominant *E. coli*, demonstrated the highest antibiotic resistant level and prevalence of integrons (integron I, 21%; integron II, 3%). Detection of phylogenetic group B2_3_ downstream of fishing villages indicates human fecal contamination as a source of *E. coli* pollution. Enteroaggregative *E. coli* (1%) were also detected immediately downstream of the fishing village. The results indicated multi-drug resistance among *E. coli* circulating in Matang estuaries, which could be reflective of anthropogenic activities and aggravated by bacterial and antibiotic discharges from village lack of a sewerage system, aquaculture farms and upstream animal husbandry.

## Introduction

*Escherichia coli* (*E. coli*), a component of the common intestinal microbiota in humans and warm-blooded animals, is an important indicator of fecal contamination in aquatic environments and food. Certain strains of *E. coli* are able to cause gastrointestinal and extraintestinal infections in humans. The pathogenic *E. coli* that cause gastrointestinal infections include enterohemorrhagic *E. coli* (EHEC), also referred to as Shiga toxin-producing *E. coli* (STEC) or verocytotoxic *E. coli* (VTEC), enteropathogenic *E. coli* (EPEC), enteroaggregative *E. coli* (EAEC), enterotoxigenic *E. coli* (ETEC), enteroinvasive *E. coli* (EIEC), and diffusely adherent *E. coli* (DAEC) (Galvin et al., 2010; Koczura et al., 2013). These pathogenic *E. coli*, together with other virulent strains of *E. coli* could cause extraintestinal infections in humans such as pyelonephritis, urinary tract infection, cystitis, neonatal meningitis, and bacteremia (Johnson and Stell, 2000; Galvin et al., 2010; Koczura et al., 2013).

*E. coli* is divided into four main phylogenetic groups A, B1, B2, and D, based on the presence and absence of, *chu*A, a gene that is responsible for heme transport in enterohemorrhagic O157:H7 *E. coli*; *yja*A, an unknown functional gene which is identified in the recent complete genome sequence of *E. coli* K-12; and TSPE4.C2, an anonymously designated DNA fragment which is the non-coding region in *E. coli* strains (Clermont et al., 2000). These four phylogenetic groups of *E. coli* appear to have distinctive genetic and phenotypic characteristics that are associated with different ecological niches (Bergthorsson and Ochman, 1998; Johnson et al., 2001; Gordon and Cowling, 2003; Gordon, 2004; Walk et al., 2007). It is interesting to note that most of the *E. coli* strains that are able to endure environmental stresses in the aquatic environment belong to the B1 phylogenetic group (Walk et al., 2007). In addition, *E. coli* strains that belong to the phylogenetic groups B2 and D harbor more virulent genes than those belonging to A and B1 (Johnson et al., 2001). The extraintestinal pathogenic *E. coli* strains are mostly phylogenetic group B2 and to a lesser extent of group D; while the commensal *E. coli* strains are commonly categorized into phylogenetic group A and B1 (Maynard et al., 2004; Vignaroli et al., 2012; Koczura et al., 2013; Pereira et al., 2013). Escobar-Páramo et al. (2004) found that most ETEC, EHEC, and EIEC belong to the phylogenetic groups A and B1; while EPEC, EAEC, and DAEC are usually not linked to any phylogenetic grouping.

The occurrence of *E. coli* in the environment, particularly in aquatic systems, is a global public health concern, especially when the water is used for human activities. The problem is recently exacerbated by the emergence of antimicrobial resistant *E. coli* strains in aquatic environments worldwide (Koczura et al., 2013; Pereira et al., 2013). Antibiotic resistant *E. coli* and other enteric bacteria, that survived the extensive antibiotic treatments in the gut of humans or animals, can enter aquatic systems through discharge from poultry and livestock production, and hospital and municipal wastewaters (Pruden et al., 2006; Pereira et al., 2013). Therefore, the rivers that are used for recreational activities, irrigation, and other purposes, can be efficient vehicles that disseminate the antibiotic resistant bacteria (Pruden et al., 2006; Su et al., 2012; Pereira et al., 2013).

Antibiotic resistance is mostly transferred among bacteria by mobile genetic elements such as integrons, plasmids and transposons. Integrons are well-organized gene expression systems that can integrate one or more non-functional gene cassettes and convert them into expressed genes (Hall and Collis, 1995; Recchia and Hall, 1995; Su et al., 2012). Integrons can easily spread the antibiotic resistance among bacterial species due to their association with plasmids. There are three types of integrons, class 1, 2, and 3 integrons and their classification is according to the integrase gene (*intI*) (Carattoli, 2003; Cambray et al., 2010; Su et al., 2012).

In addition to their use in human medicine, antibiotics are also widely used as therapeutics, prophylactics and metaphylactics in animals and aquaculture farms (Jaime et al., 2012). Twenty different antibiotics (e.g., erythromycin, tetracycline, oxalinic acid, oxytetracycline, amplicillin, norfloxacine, trimethoprim, sulfudimethoxine) and chemicals (e.g., calcium carbonate, potassium permanganate, formalin, and thiodane) are commonly used in aquaculture farms in Malaysia (Keh, 2003). Generally, antibiotics are mixed with feeds, or dissolved directly in water. However, fish are not able to metabolize the antibiotics, which are largely returned to the environment via faces that pose a potential threat to public health (Burridge et al., 2010; Jaime et al., 2012).

Several estuaries within Matang Mangrove Forest Reserve (MMFR), Perak were selected as study sites in this study. The MMFR bears the distinctive features of being one of the best managed mangrove forests in the world, an important nursery area sustaining the country's largest fisheries landings and site to the state's largest brackish water aquaculture farms (Ariffin and Nik Mustafa, 2013). The MMFR exemplifies significant economic benefits and ecological services that could be obtained through sustained forest management. However, the Matang area also demonstrates the increasing dilemma of multiple-use conflicts of land and water resources (Chong et al., 2010). Twenty eight of the 34 permanent settlements in the MMFR are fishing villages sited along the mangrove estuaries with a total population of 31,800 people (Lim and Mohamad Parit, 2001).

A recent study shows that the Matang mangrove estuaries may be facing a great challenge of anthropogenic pollution due to contamination by various potentially pathogenic bacteria including high counts of *E. coli* in both water and sediment (Ghaderpour et al., 2014). This kind of anthropogenic pollution could become a more significant public health issue if these bacteria are resistant to antibiotics. Therefore, in this study, we aimed to (i) investigate the genetic diversity of *E. coli* in Matang estuaries, (ii) determine the virulence factors of the estuarine *E. coli* isolates, and (iii) detect antibiotic resistant *E. coli* and their resistance mechanisms in Matang mangrove estuaries.

## Materials and methods

### Study sites and sample collection

**Figure 1:**
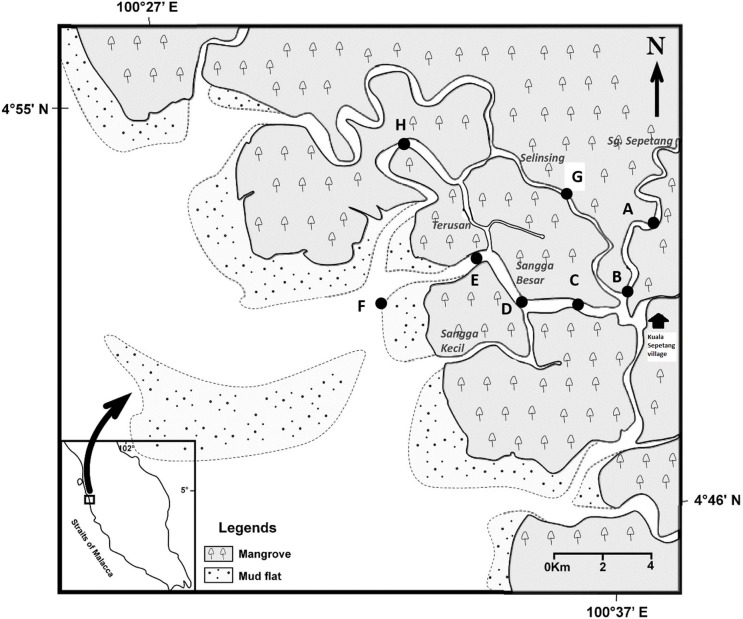
**Locations of eight sampling stations (A–H) along Sepetang, Sangga Besar and Selinsing rivers in Matang Mangrove Forest Reserve, Perak, Malaysia**.

The MMFR is located in the northwest coast of peninsular Malaysia (Figure 1). It is a silviculture production forest of 41,000 ha managed on a sustainable basis since 1902. Within the sheltered waterways of the forest reserve, nearly 8000 floating fish cages are present, while 385 shrimp and fish ponds cover a total area of 223 ha on the landward margin of the mangrove forests (Ariffin and Nik Mustafa, 2013). Cockle culture covers an extensive area of 4726 ha within and outside the Matang estuaries.

Water and sediment samplings were carried out in the various estuaries, particularly the Sangga Besar, Sepetang and Selinsing Rivers. Sangga Besar River is the main waterway (8 km) traversed by fishing boats between the fishing village at Kuala Sepetang and the coastal fishing grounds. Unlike the Sangga Besar, the Selinsing River is a long waterway (18 km) relatively less used by boats as well as having few floating fish farms at its river mouth. Eight sampling sites (A–H) were set up, from upstream to downstream of the estuary (Figure 1). Station A is situated at the upper estuary of the Sepetang River, while stations B to F are located along the Sangga Besar River. Two further sampling sites, G and H were set up at the upstream and downstream of the Selinsing River, respectively. Station A is located upstream of the Kuala Sepetang village, while Station B, C, and G are situated 1–7 km downstream of the village, receiving the untreated sewage and other anthropogenic pollution from the village. Station D and E are located near to cockle culture beds and fish cages, respectively. Station F is situated at the river mouth downstream of the fish cages close to other cockle culture areas on the coastal mudflat. Station H is located 15 km downstream from the Kuala Sepetang village on Selinsing River, with very little human activity (Figure 1).

Four water and sediment samples were collected at each sampling station in October 2011 (wet season) and May 2012 (dry season). Water samples (0.5 m depths) were collected in acid washed bottles. A 15 cm × 15 cm Ekman grab (Wildco, USA) was used to collect bottom sediment. The top 5 cm surface of the collected sediment samples was sampled using a spatula and kept in sterile bottles. All samples were stored in ice, and immediately transferred into the laboratory freezer kept at 4°C until the analyses were carried out within 24 h of sampling.

### Isolation of *E. coli* and detection of their housekeeping and virulence genes

Membrane filtration technique was used to isolate *E. coli*. Water (5 ml) and sediment (1 g in 9 ml of sterile 0.85% saline) samples were filtered through 0.45 mm nitrocellulose filters (47 mm diameter). The filters were then transferred on CHROMagar ECC (CHROMagar Inc., Paris, France) and incubated at 42 ± 0.5°C for 24 h. Blue colonies as presumptive *E. coli* were randomly picked and purified on Luria Bertani medium. After purification, presumptive *E. coli* isolates were kept in stab and in glycerol forms (Luria Bertani Broth with 50% glycerol) at 37°C and −20°C, respectively for the future analysis. The identity of the presumptive *E. coli* isolates was confirmed by PCR targeting the *pho*A gene, an *E. coli* housekeeping gene (Yu and Thong, 2009).

Two different multiplex PCR assays were carried out for all confirmed *E. coli* to identify the pathogenic *E. coli* (EHEC, EPEC, EAEC, ETEC, EIEC and DAEC). For both multiplex PCR, reaction mixture was carried out in 25 μL volume consisting of 1X green buffer (5X green GoTaq reaction buffer, pH 8.5), 0.5U of *Taq* DNA polymerase (Promega, Madison,Wis, USA), 1.65 mM MgCl_2_, 0.3μM of the selected primers, 220 μM of each deoxynucleoside triphosphate (dNTP), and 5 μL of DNA template. PCR amplification and primers used in this study were previously described by Gómez-Duarte et al. (2009). Primers sequences are listed in Supplementary Table 1. Six positive control strains (*E. coli* 2060–004, E2348/69, JM221, E9034A, C1845, and EC-12) were used in this study and sterile water was included as negative control. The six positive strains were provided by Oscar G. Gomez-Duarte, International Enteric Vaccines Research Program [IEVRP], University of Iowa Children's Hospital, Iowa City, IA (Gómez-Duarte et al., 2009).

### Phylogenetic grouping

A triplex PCR assay was used to determine the phylogenetic groups (A, B1, B2, and D) of *E. coli*. PCR conditions for presence or absence of *chu*A, *yja*A, and TSPE4-C2 were used as described by Clermont et al. (2000) and Carlos et al. (2010) (Table 1). Each reaction was performed in 25 μL volume containing 5 μL (appr 10 ng) DNA template, 120 μM each dNTP, 1X PCR buffer, 0.5 U *Taq* polymerase, 1.5 mM MgCl_2_, 0.4 μM of yjaA primers, 0.24 μM each chuA, and TSPE4-C2 primers (Supplementary Table 1). Combination of presence or absence of *chu*A, *yja*A, and TSPE4-C2 was used for identification of phylogenetic grouping as follows: phylogenetic group A (A0, −∕−∕−; A1, −∕+∕−), phylogenetic group B (B1, −∕−∕+), phylogenetic group B2 (B2_2_, +∕+∕−; B2_3_, +∕+∕+) and phylogenetic group D (D1, +∕−∕−; D2, +∕−∕+) (Carlos et al., 2010).

**Table 1:** **Antibiotic susceptibility characterization of 148 isolates of *E. coli* isolated from estuarine water and surface sediments of Matang mangrove estuary, Perak, Malaysia**.

**Characteristics**	**No. of Isolates**	**Antibiotics^a^^,^^b^^,^^c^**															**Antibiotic resistance pattern**				
		**I**				**II**				**III**				**IV**	**V**	**VI**					
		**N**	**S**	**K**	**CN**	**AMC**	**AMP**	**CRO**	**EFT**	**ENR**	**CIP**	**OA**	**NA**	**TE**	**C**	**SXT**	**≥1**	**≥3**	**≥6**	**≥9**	**All**
																	**antibiotic**	**antibiotics**	**antibiotics**	**antibiotics**	**antibiotics**
**STATIONS**																					
A	17	14 (82)	7 (41)	3 (18)	2 (12)	1 (6)	3 (18)	1 (6)	1 (6)	3 (18)	1 (6)	2 (12)	3 (18)	6 (35)	2 (12)	1 (6)	16 (94)	7 (41)	2 (12)	1 (6)	1 (6)
B	28	24 (86)	12 (43)	9 (32)	1 (4)	4 (14)	16 (57)	0 (0)	0 (0)	11 (39)	7 (25)	14 (50)	14 (50)	13 (46)	10 (36)	13 (47)	28 (100)	15 (54)	13 (47)	8 (29)	3 (11)
C	8	5 (63)	2 (25)	1 (13)	0 (0)	0 (0)	2 (25)	0 (0)	0 (0)	3 (38)	1 (13)	3 (38)	3 (38)	3 (38)	1 (13)	0 (0)	6 (75)	3 (38)	2 (25)	1 (13)	0 (0)
D	24	13 (54)	12 (50)	5 (21)	0 (0)	0 (0)	10 (42)	0 (0)	0 (0)	7 (29)	3 (13)	3 (13)	5 (21)	9 (38)	8 (33)	6 (25)	19 (79)	9 (38)	6 (25)	5 (21)	0 (0)
E	20	16 (80)	6 (30)	2 (10)	0 (0)	0 (0)	3 (20)	0 (0)	0 (0)	1 (5)	0 (0)	0 (0)	1 (5)	1 (5)	1 (5)	0 (0)	18 (90)	1 (5)	0 (0)	0 (0)	0 (0)
F	8	7 (88)	2 (25)	2 (25)	1 (13)	5 (63)	3 (38)	0 (0)	2 (25)	2 (25)	0 (0)	1 (13)	2 (25)	2 (25)	0 (0)	1 (13)	7 (87)	4 (50)	2 (25)	2 (25)	0 (0)
G	19	14 (74)	2 (11)	4 (21)	0 (0)	6 (32)	8 (42)	1 (5)	1 (5)	3 (16)	1 (5)	3 (16)	4 (21)	6 (32)	2 (11)	5 (26)	17 (90)	9 (48)	3 (16)	1 (5)	0 (0)
H	24	16 (67)	3 (13)	2 (8)	1 (4)	0 (0)	2 (8)	0 (0)	0 (0)	2 (8)	1 (4)	1 (4)	1 (4)	2 (8)	0 (0)	2 (8)	17 (71)	2 (8)	1 (4)	1 (4)	0 (0)
**PHYLOGROUPES**																					
A	38	29 (76)	10 (26)	8 (21)	2 (5)	5 (13)	16 (42)	1 (3)	1 (3)	13 (34)	5 (13)	11 (29)	11 (29)	12 (32)	8 (21)	7 (18)	34 (98)	15 (40)	11 (29)	6 (16)	1 (3)
B1	65	50 (77)	14 (22)	16 (25)	1 (2)	6 (9)	22 (34)	0 (0)	1 (2)	17 (26)	8 (12)	15 (23)	20 (31)	21 (32)	13 (20)	14 (22)	55 (85)	25 (39)	15 (23)	11 (17)	3 (5)
B2	7	3 (43)	5 (71)	0 (0)	0 (0)	2 (29)	1 (15)	0 (0)	0 (0)	0 (0)	0 (0)	0 (0)	0 (0)	2 (29)	0 (0)	1 (15)	6 (86)	2 (29)	0 (0)	0 (0)	0 (0)
D	38	27 (71)	10 (26)	4 (11)	2 (5)	3 (8)	9 (24)	1 (3)	2 (5)	2 (5)	1 (3)	1 (3)	2 (5)	7 (18)	3 (8)	3 (8)	34 (98)	8 (21)	3 (8)	2 (5)	0 (0)
**SEASONAL**																					
Wet	56	42 (75)	2 (4)	6 (11)	0 (0)	0 (0)	14 (25)	0 (0)	0 (0)	3 (5)	3 (5)	4 (7)	5 (9)	5 (9)	5 (9)	3 (5)	44 (79)	5 (9)	3 (5)	3 (5)	0 (0)
Dry	92	67 (73)	44 (48)	22 (24)	5 (5)	16 (17)	34 (37)	2 (1)	4 (4)	29 (32)	11 (12)	23 (25)	28 (30)	37 (40)	19 (21)	25 (27)	84 (91)	45 (49)	26 (28)	16 (17)	4 (4)
Total	148	109 (74)	46 (31)	28 (19)	5 (3)	16 (11)	47 (33)	2 (1)	4 (3)	32 (22)	14 (10)	27 (18)	33 (22)	42 (28)	24 (16)	28 (19)	128 (87)	50 (34)	29 (20)	19 (13)	4 (3)

a Number of E. coli isolates (percentage over number of isolates,%).

b Antibiotic class. I, aminoglycosides; II, beta-lactams; III, quinolones/fluoroquinolones; IV, tetracyclines; V, phenicols; VI, sulpha group.

c Antibiotic. N, neomycin; S, streptomycin; K, kanamycin; CN, gentamicin; AMC, amoxicillin/clavulanic acid; AMP, ampicillin; CRO, ceftriazone; EFT, ceftiofur; ENR, enrofloxacin; CIP, ciprofloxacin; OA, oxolinic acid; NA, nalidixic acid; TE, tetracycline; C, chloramphenicol; SXT, sulfamethoxazole/trimetroprim.

### Antibiotic susceptibility testing

Agar disc diffusion method on Mueller-Hinton Agar (Oxoid, Basingstoke, UK) was used to determine the antibiotic resistance patterns among *E. coli* isolates. A total of 15 antibiotics belonging to 7 classes were tested, including aminoglycosidase: gentamicin (CN, 10 μg), kanamycin (K, 30 μg), streptomycin (S, 25 μg), and neomycin (N, 30 μg); β-lactams: amoxicillin/clavulanic acid (AMC, 30μg), ampicillin (AMP, 10 μg), ceftriaxone (CRO, 30 μg), ceftiofur (EFT, 30 μg); quinolones: nalidixic acid (NA, 30 μg), oxolinic acid (OA, μg); fluoroquinolone: enrofloxacin (ENR, 5 μg), ciprofloxacin (CIP, 5 μg); tetracycline: tetracycline (TE, 30 μg); phenicols: chloramphenicol (C, 30 μg), and sulfamethoxazole/trimethoprim (SXT, 25 μg) (Oxoid Ltd.,Basingtoke, Hampshire, England). The bacteria were designated as resistant, intermediate and sensitive according to the Clinical Laboratory Standards Institute (CLSI)'s recommendations (CLSI, 2012). Bacteria strains that were resistant and intermediate were categorized as non-susceptible while the sensitive strains were categorized as susceptible.

### Detection of class 1, 2, and 3 integrons

All *E. coli* isolates were screened for integrase genes. Three different sets of primers were used to detect class 1, 2, and 3 integrase genes (Supplementary Table 1). Primers IntI1-F, IntI1-R and 5′CS and 3′CS were used to detect *intI*1 gene and class 1 integron gene cassettes, respectively. For class two integrons, IntI2-F, IntI2-R, and attI2-F/orfX-R primers were used for amplifying *intI*2 and class 2 integron gene cassettes, respectively. The primer IntI3-F/IntI3-R was used to amplify *intI*3 gene.

The reaction mixture for all PCR assays was performed in 25 μL volume which consisted of 1X PCR green buffer, 1.4 mM MgCl_2_, 120 μM of each dNTP, 0.06 μM of each primers, and 0.5 U of *Taq* DNA polymerase (Promega, Madison, Wisconsin, USA). The multiplex PCR amplification of integrase type 1 and 2, and class 1 integron gene cassettes was performed as previously described by Lim et al. (2009).

### Confirmation of PCR products by DNA sequencing

PCR products were purified and submitted to a commercial company (First BASE Laboratories, Malaysia) for sequencing using BigDye® Terminator v3.1 cycle sequencing kit chemistry. Homologs search against the GenBank databases of nr/nt using BLASTN (http://blast.ncbi.nlm.nih.gov/blast) were carried out for all the DNA sequences obtained.

### Genetic diversity of *E. coli* isolates using REP-PCR

Genetic diversity of 148 *E. coli* isolates was analyzed by Repetitive Extragenic Palindromic-PCR (REP-PCR) using REP primer (Supplementary Table 1). Bacterial isolates were grown in 1 ml of sterile Luria Bertani broth overnight at 37°C. Bacterial suspension in 1.5 ml microfuge tubes were centrifuged and washed with sterile Phosphate buffered saline (PBS) and 1X TE (Tris-EDTA) buffer. Bacterial pellets were suspended in sterile water and were then heated at 99°C for 10 min and chilled on ice for 20 min. The extracted crude DNA was used for amplification with REP oligonucleotides (Operon Biotechnologies GmBH, Germany) as previously reported (Lim et al., 2009). REP-PCR assay was carried out in 25 μL volume which included of 1X buffer (5X buffer GoTaq reaction buffer, pH 8.5), 1U of *Taq* DNA polymerase (Promega, Madison, Wisconsin, USA), 2.5 mM MgCl_2_, 200 μM of each dNTP 0.5 μM of the primer and 4 μL of DNA template. Temperature program for this assay consisted of initial denaturing at 94°C for 7 min, followed by 30 cycles of 30 s at 94°C, 1 min at 44°C, 8 min at 72°C and a final extension step 16 min at 65°C. PCR products were loaded in 1.5% agarose gels and stained in Gel red nucleic acid stain (Biotium Inc, USA). Banding patterns were analyzed by GelCompar II, version 2.5 (Applied Maths, Kortrijk, Belgium).

### Statistical analysis

Prevalence of the antibiotic resistance was determined using x/y 100% equation, where x is defined as number of resistant *E. coli* isolates to antibiotics, and y is total isolated number of *E. coli* from each sample. Principle component analysis (PCA) was used to depict the distribution of *E. coli* with respect to phylogenetic groups, integron genes, and antibiotics resistance among eight sampling stations in Matang mangrove estuary. To perform the PCA procedure, the number of isolates of *E. coli* containing the phylogenetic groups, integron genes and antibiotics resistance was square root transformed to meet the parametric assumptions. PCA was performed using CANOCO 4.5 software (Ter Braak and Smilauer, 2002). Pearson's chi-squared goodness-of-fit test was applied to evaluate the significant difference among phylogenetic groups based on the frequency data. Also, comparisons of associations between phylogroups and antibiotic resistance were done using the chi- square test when no more than 20% of the expected cell frequencies were < 5 and all individual expected counts were ≥1, otherwise Fisher's exact test (two-tailed) was used. The degree of association between the presence of antibiotic resistance and presence of integron genes were tested using the correlation (R_n_) of Ives and Gibbons for dichotomous normal-scale data in Zar (2010). Significance was tested using the chi-square contingency test. All tests were performed at the 5% level of significance.

## Results

### Distribution of phylogenetic groups and pathotypes of *E. coli* in matang mangrove estuaries

A total of 175 presumptive *E. coli* bacteria were isolated from 64 water and sediment samples, during dry and wet seasons. Out of 175 presumptive *E. coli* isolates, 148 (85) were confirmed as *E. coli*, of which 17 were from station A, 28 from station B, 8 from station C, 24 from station D, 20 from station E, 8 from station F, 19 from station G and 24 from station H.

**Figure 2:**
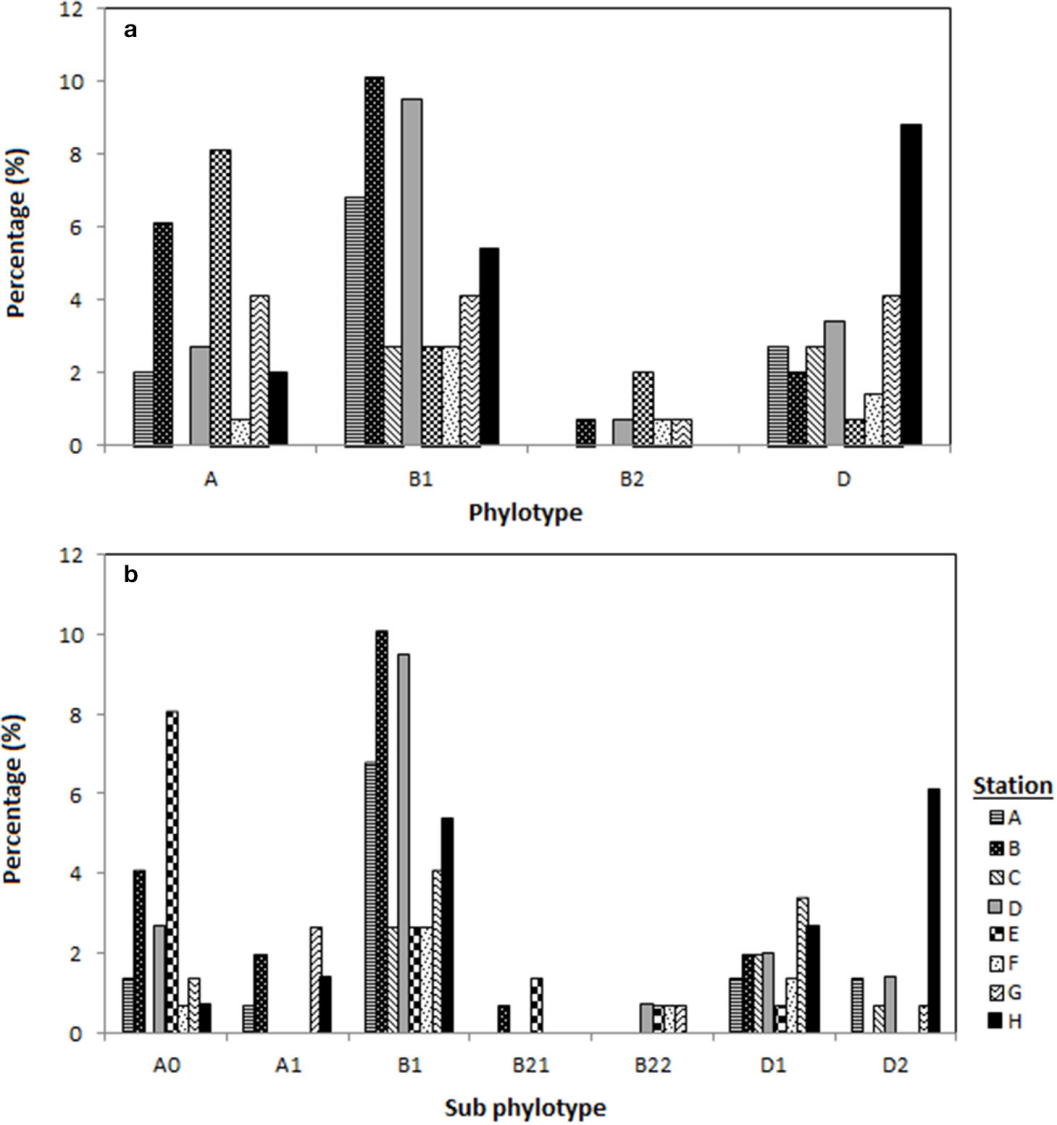
**Distribution of the *E. coli* phylogenetic groups in eight different stations located in Sepetang River (A), Sangga Besar River (B–E) and Selinsing River (G and H) and coastal waters (F), (a) distribution of *E. coli* according to the respective phylogenetic groups (A, B1, B2, D) and (b) according to phylogroup subtyping (A0, A1, B1, B21, B22, D1, D2)**.

Among the 148 *E. coli* isolates, phylogenetic group B1 was the most prevalent (*n* = 65, 44%) followed by phylogenetic group A and D which had similar prevalence (*n* = 38; 26%). The phylogenetic group B2 was the least prevalent (5%) in Matang mangrove estuaries, and was only detected during the dry season. Phylogenetic groups B1 and D were present at all the stations located along Sangga Besar and Selinsing Rivers. Phylogenetic group B1 was the most frequent isolate from all stations except station E and H, where phylogenetic groups A and D predominated, respectively (Figure 2). Phylogenetic group D was more frequently isolated in the wet season (32%) than in the dry season (22%). The prevalence of group A was significantly higher at station E, located in Sangga Besar River (χ^2^ = 16.526, *df* = 6, *p* = 0.011); while station E recorded a significantly lower occurrence of *E. coli* subset D. Station H located downstream of Selinsing River had significantly higher occurrence of *E. coli* subset D (χ^2^ = 20.105, *df* = 6, *p* = 0.005) (Figures 1, 2).

The genotypes of each phylogenetic group (A0/A1, B22/B23, and D1/D2) were not distributed homogenously among all stations. Genotype A0 comprised almost two thirds of phylogenetic group A and could be detected in almost all stations, while genotype A1 was only present in the upstream of Sangga Besar River (station A and B) and Selinsing River (station G and H). Genotype B2_3_ was only detected from station D, E, F, and G which are all located downstream of a fishing village (Figure 2b).

None of the 148 isolates was EHEC, EPEC, ETEC, DAEC, or EIEC. However, two isolates originated from station D located upstream of Sangga Besar River and station G located upstream of Selinsing River were identified to be EAEC.

### Antibiotic resistance among the *E. coli* isolates

Among the 148 *E. coli* isolates from Matang mangrove estuaries, 128 (87%) were non-susceptible to at least one of 15 antibiotics tested (Table 2). Only 20 (14%) *E. coli* isolates were sensitive to all the 15 tested antibiotics. The antibiotic resistance percentages were: N (74%), S (31%), K (19%), CN (3%), AMC (11%), AMP (33%), CRO (1%), EFT (3%), ENR (22%), CIP (10%), OA (18%), NA (22%), TE (28%), C (16%), and SXT (19%) (Table 1 and Supplementary Table 2).

**Table 2:** **Intergron and gene cassette detection in Matang estuary**.

**Characteristic**		**Isolates no**	**None integras gene**	**IntI1**	**IntI2**	**IntI1 & IntI2**	**Gene cassette**	**Contents of gene cassette**
**STATIONS**								
A		17	16 (94)	1 (6)	1 (6)	1 (6)	0 (0)	
B		28	15 (54)	13 (46)	2 (7)	2 (7)	2 (7)	aadA22; dfrA17
C		8	7 (88)	1 (13)	0 (0)	0 (0)	0 (0)	
D		24	17 (71)	7 (29)	1 (13)	1 (13)	2 (8)	aadA1; dfrA5
E		20	20 (100)	0 (0)	0 (0)	0 (0)	0 (0)	
F		8	5 (63)	3 (38)	0 (0)	0 (0)	0 (0)	
G		19	15 (79)	4 (21)	0 (0)	0 (0)	2 (11)	dfrA17; dfrA5
H		24	22 (92)	2 (8)	0 (0)	0 (0)	1 (4)	dfrA1+aadA1
**PHYLOGROUPES**								
A		38	29 (76)	9 (24)	3 (8)	3 (8)	1 (3)	dfrA1+aadA1
B1		65	50 (77)	15 (23)	1 (2)	1 (2)	2 (3)	dfrA5; aadA22
B2		7	6 (86)	1 (14)	0 (0)	0 (0)	0 (0)	
D		38	32 (84)	6 (16)	0 (0)	0 (0)	4 (11)	dfrA5; aadA1; dfrA17; dfrA17
**SEASONAL**								
Dry		56	53 (95)	3 (5)	0 (0)	0 (0)	0 (0)	
Wet		92	64 (70)	28 (31)	4 (4)	4 (4)	7 (8)	aadA22; dfrA17; aadA1; dfrA5;dfrA17; dfrA5; dfrA1+aadA1
Total		148	117 (79)	31 (21)	4 (3)	4 (3)	7 (5)	

**Figure 3:**
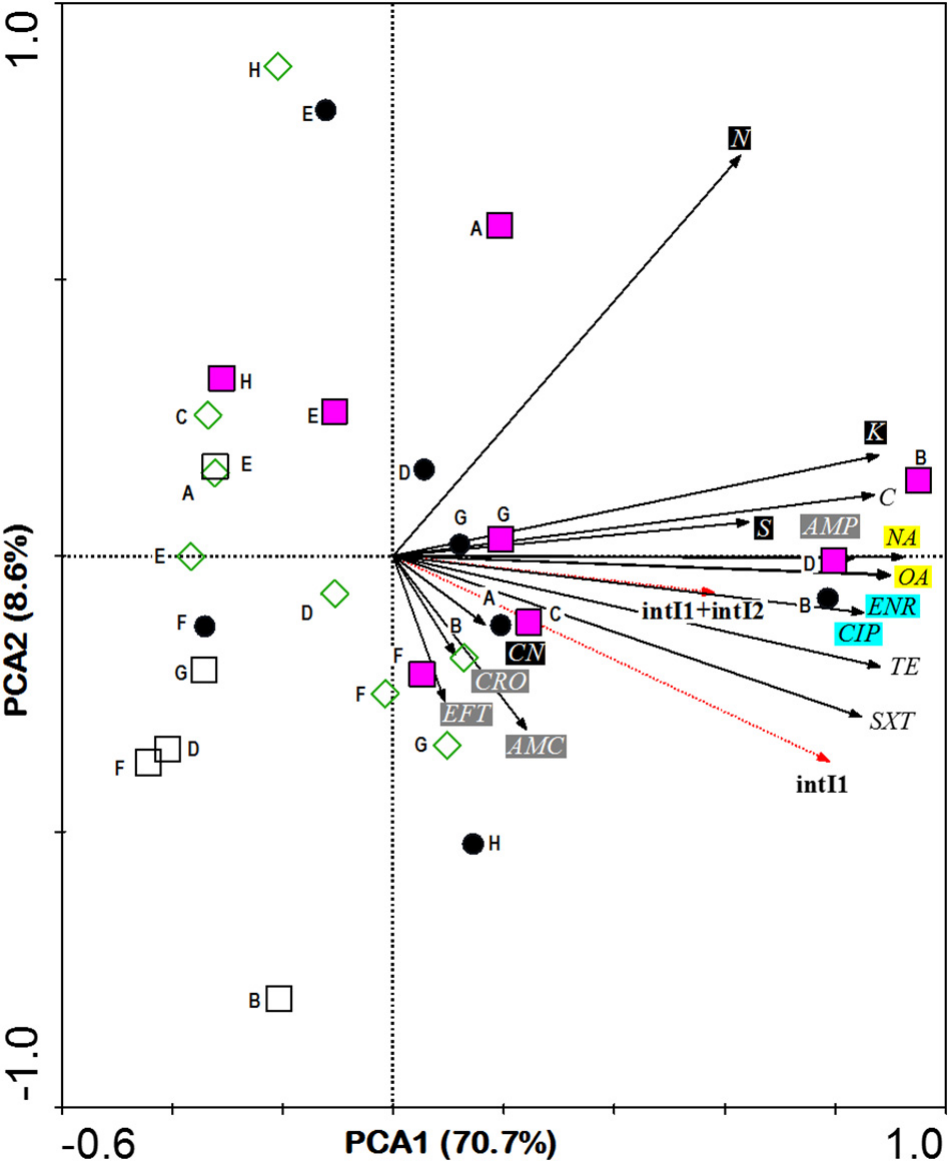
**PCA ordination biplot showing the distribution of *E. coli* phylogenetic groups by sampling stations with respect to their resistance to various antibiotics and the integras genes they carried**. The first (PCA1) and second (PCA2) principal components respectively explained 70.7 and 8.6% of the total variance of the explanatory variables. Phylogroups are denoted by various symbols: black circle A, pink square B1, empty square B2 and empty diamond D. Bold letter beside symbol denotes sampling stations located in Sepetang River (A), Sangga Besar River (B–E), Selinsing River (G and H) and adjacent coastal water (F). Black solid arrows indicate isolates resistant to 15 antibiotics: Class I (black box), CN, aminoglycosidase: gentamicin; K, kanamycin; S, streptomycin; N, neomycin; Class II (gray box); AMC, β-lactams: amoxicillin/clavulanic acid; AMP, ampicillin; CRO, ceftriaxone; EFT, ceftiofur; Class III (blue box); ENR, fluoroquinolone: enrofloxacin; CIP, ciprofloxacin; Class IV (yellow box); NA, quinolones: nalidixic acid; OA, oxolinic acid; Class V; TE, tetracycline: tetracycline; Class VI; C, phenicols: chloramphenicol and Class VII; SXT, sulfamethoxazole/trimethoprim. Red dotted arrows indicate *E. coli* integron gene (s).

In comparisons among stations, Principle Component Analysis (PCA) showed that high antibiotic resistance among the *E. coli* isolates was detected at the stations (B and D) near to the fishing village (positive direction of PCA1, Figure 3). Most *E. coli* isolated from these stations were phylogenetic group B1 and carried class 1 integron. Moreover, high antibiotic resistances especially to aminoglycosides and beta-lactams were also detected in *E. coli* isolates from station F located on the cockle mud flat. On the other hand, low antibiotic resistance of *E. coli* isolates was detected at the stations with less anthropogenic influence (station H) and close to the fish cages (station E) (Table 1 and Figure 3). Overall, *E. coli* isolates from Matang mangrove estuaries demonstrated high prevalence of multi-drug resistance (MDR). One third of the tested *E. coli* isolates (34%) were found to be non-susceptible to three or more antibiotics (3R); and about one in every five isolates (20%) were non-susceptible to six or more antibiotics (6R) tested in this study (Table 1 and Supplementary Table 2). Further, four out of 148 isolates (3%) were non-susceptible to all of the 15 antibiotics examined in this work (Table 1 and Supplementary Table 2). Fifty out of 148 (34%) *E. coli* samples isolated from Matang mangrove estuaries demonstrated resistance to at least three antibiotic classes and 10% were non-susceptible to all the seven antibiotic classes tested (Table 1 and Supplementary Table 2).

The analysis of the phylogenetic groups with respect to their antibiotic resistance indicated that all groups showed some level of resistance to antibiotics. However, *E. coli* isolates in A and B1 phylogenetic groups (positive direction of PCA1) generally shows increasing resistance to most antibiotics as compared to the B2 and D phylogenetic groups (negative direction of PCA1). The phylogenetic group A and B1 demonstrated higher non-susceptibility level toward beta-lactams, (fluoro) quinolones, tetracycline, phenicols, and sulfamethoxazole/trimethoprim. Additionally, multi-drug resistance phenotype was more frequently identified in groups A and B1 (Table 1 and Supplementary Table 2). Chi-square independence test found only significant association between phylogroups with enrofloxacin (ENR; χ^2^ = 12.27; *df* = 3; *p* = 0.007), oxolinic acid (OA; χ^2^ = 11.71; *df* = 3; *p* = 0.008) and nalidixic acid (NA; χ^2^ = 12.04; *df* = 3; *p* = 0.001), which are all (fluoro) quinolones.

### Prevalence of the class 1, 2, and 3 integrons

All 148 *E. coli* isolates were screened for class I, II and III integrons. Class I and II integrons were detected in 31 (21%) and 4 (3%) isolates, respectively (Table 2). Class III integron was absent in the *E. coli* isolates in Matang mangrove estuaries. Four isolates (3%) harbored both class I and II integrons. Of the 31 *intI*1-positive isolates, only 7 (23%) harbored gene cassettes; while none of the *intI*2 positive isolates carried any gene cassette. The size of the gene cassette in class 1 integron ranged from 700 to 1200 bp. Only one out of 148 isolates was found to carry more than one gene cassette, and this particular isolate was obtained from downstream of the Selinsing River (station H). The DNA sequences of all class I integrons have been deposited in GenBank under accession nos. KR952338-KR952345.

Five types of the resistance gene cassettes were detected in this study (Table 2). Three types of *dfr*A (*dfr*A 1, *dfr*A 5, and *dfr*A 17) were identified, which encoded the dihydrofolate reductase enzyme, mediating resistance to trimethoprim. Additionally, two types of the *aad*A (*aad*A 1 and *aad*A 22) were detected in class 1 integron that confers resistance to spectinomycin and streptomycin. The *aad*A and *dfr*A genes can be present alone or in combination with each other and other resistance genes as only one cassette combination (*dfr*A1 + *aad*A1) was recovered in this study (Table 2). All *E. coli* isolates that carried the *aad*A and *dfr*A genes were resistant to aminoglycosides and SXT, respectively (Table 2).

Half of the *intI* positive *E.coli* belonged to group B1 (*n* = 15, 48%) followed by nine isolates (29%) in group A and six isolates (19%) in group D. Only one isolate in group B2 was detected as *intI*1 positive. This integron gene was closely associated with most of the antibiotic resistance. In the PCA biplot, the gradients of increasing number of *IntI* positive isolates were in the same direction as the gradients of antibiotic resistance (Figure 3). Resistance to all antibiotics (except N, AMC, CN, and CRO), i.e., 73.3% of the 15 tested antibiotics, was significantly correlated to *intI*1-positive isolates (*p* < 0.001), with correlation (R_n_) values that ranged from 0.50 (S) to 0.81 (SXT) (Supplementary Table 3). Of the eleven antibiotic resistant isolates that were *intI*1-positive, seven were also significantly correlated to *intI*2-positive isolates (*p* < 0.03), with R_n_ values that ranged from 0.49 (TE) to 0.73 (C). Among four *intI*2 positive isolates, three and one isolates belonged to phylogenetic group A and B1, respectively. Moreover, four out of seven isolates (57%) harbored gene cassette class 1 belonged to phylogenetic group D (Table 2).

### Genetic diversity of *E. coli*

All the confirmed 148 *E. coli* isolates that were recovered in October 2011 and May 2012 were subjected to REP-PCR. REP-PCR was carried out to determine the diversity of *E. coli* using UPGMA (unweighted pair-group method using arithmetic averages). REP-PCR subtyped the 148 *E. coli* isolates into 118 different REP profiles comprising of 200 to 2000 bp DNA fragments. At 80% similarity, the coefficient of similarity (*F*-value) ranged from 0.69 to 1.0 and the 148 isolates were clustered into seven groups (Supplementary Table 4). Cluster I and V included 76 and 27 *E. coli* isolates from different stations, respectively. Another 45 *E. coli* isolates were grouped into five clusters with each comprising of two to 18 isolates. Most of resistant isolates including MDR phenotypes were grouped into cluster, I, V, and III of the REP-PCR profile (Supplementary Table 4).

## Discussion

### Multi-drug resistant (MDR) *E. coli* in matang mangrove estuaries

In the present study, the Matang mangrove estuaries harbor a high prevalence of antibiotic resistant *E. coli* in surface waters and sediments., the results indicated that four in every five *E. coli* isolates from Matang estuaries were non-susceptible to one or more antibiotics tested (1R); and one out of four 1R Isolates was non-susceptible to six or more antibiotics tested (Table 1). About one-third of the *E. coli* isolated from Matang estuaries were MDR and this figure is relatively higher compared to Tagus estuary in Portugal, in which 19% of the *E. coli* isolates were MDR (Pereira et al., 2013). However, the antibiotic resistant level was lower compared to the 88% found in Dongjiang River, China (Su et al., 2012). Laroche et al. (2009) also reported a relatively high level of MDR *E. coli* (39%) in Seine River, France. The high level of MDR *E. coli* observed in all of these studies from different parts of the world, including the current work highlighted the potential risk of dissemination of antibiotic resistance traits via the aquatic system.

PCA analysis showed that the prevalence of antibiotic resistance in *E. coli* was related to the sampling stations, in which antibiotic resistant *E. coli* was more frequently isolated from the upstream of both Sangga Besar and Selinsing Rivers. Station B was one of the stations with high numbers of MDR *E. coli* isolates recovered. This is likely due to its proximity to Kuala Sepetang fishing village where there is open sewage disposal and poor sanitation (Ghaderpour et al., 2014). Majority of *E. coli* isolates that demonstrated 100% non-susceptibility to all 15 antibiotics tested were isolated from station B. Also, *E. coli* isolates that showed non-susceptibility toward aminoglycosides and beta-lactams were more frequently isolated from stations located downstream of Kuala Sepetang or between the latter and Sangga Besar fishing village at the river mouth (Table 1). The findings suggested anthropogenic sources as the major contributor to the presence of antibiotic resistant *E. coli* in Matang mangrove estuaries. As the results show, beta-lactams resistant *E. coli* (i.e., non-susceptible to amocixillin-clavulanic acid, ampicillin, ceftriazone, or ceftiofur) was least frequently isolated from station H, but more frequently isolated from station F (75%) and station B (57%) located downstream of the villages. The highest antibiotic resistance prevalence was detected for neomycin (74%), ampicillin (33%), streptomycin (31%), and tetracycline (28%). The major antibiotic resistance found among *E. coli* isolates was in agreement with previous studies in aquatic environments (Hamelin et al., 2007; Laroche et al., 2009; Pereira et al., 2013). This resistance may reflect the abuse of antibiotics in human and veterinary medicine and aquaculture farms in the study area. The direct discharge of untreated sewage from the villages into the estuarine system, active fish and shrimp farming, as well as the presence of upstream swine farms are the main contributing factors to the high prevalence of MDR *E. coli* in Matang estuaries. Aquaculture could be a significant contributor to antibiotic resistance since the use of antibiotics, pesticides, and other chemotherapeutic agents in feed additives and treatment baths is as yet unregulated in Malaysia (Majusha et al., 2005; Ibrahim et al., 2010). Sulfonamides, tetracyclines, oxytetracycline, ampicillin, chloramphenicol, and quinoline are common antibiotics used in aquaculture farms in Perak as well as in the country (Mohamed et al., 2000; Keh, 2003). Nevertheless, reservoir sources of *E.coli* may include shorebirds and other feral animals such as monkeys, flying foxes and bats, as well as domestic animals, especially dogs (Frenzel and Couvillion, 2002).

It is interesting to note that even though the *E. coli* count at station F was low as reported by Ghaderpour et al. (2014), which resulted in lower isolation rate, a high level of antibiotic resistance was found in *E. coli* isolates from station F (Table 1 and Figure 2). A high bacteria count is expected at the river mouth due to bacterial contamination from the upstream, but due to the fact that *E. coli* is sensitive to high salinity (Gao et al., 2013), at station F, most of the *E. coli* would have been inactivated except antibiotic resistant strains that are also tolerant to high salinity. On the other hand, low antibiotic resistance was found in station H, downstream of Selinsing River, despite the high count of *E. coli* reported previously (Ghaderpour et al., 2014). Since station H is far away from obvious anthropogenic sources, its high count of *E. coli* could originate from wildlife. It has been shown in other studies that the prevalence of antibiotic resistance was low in wild animals compared to human and agricultural sources (Sayah et al., 2005; Edge and Hill, 2007).

### Distribution of class I and II integrons among *E. coli* from matang mangrove estuaries

Integron is an important factor in developing MDR resistance phenotypes among *E. coli* and other bacterial species since they are able to transfer different resistance genes simultaneously (Martinez, 2009). In the present study, 21% of *E. coli* isolates were integron positive. It was higher than those values reported in previous studies, 11% in France and Portugal (Laroche et al., 2009; Pereira et al., 2013), and 15% in Czech Republic (Dolejská et al., 2009). The frequency of the class 2 integron (3%) among *E. coli* isolates in Matang estuaries concurred with earlier studies in estuary (Laroche et al., 2009), in animal and human *E. coli* isolates (Kang et al., 2005; Skurnik et al., 2005; Cocchi et al., 2007). No class 3 integron was found in this study since integron class 3 is rare even among the human and animal *E. coli.* This finding is consistent with other reports (Laroche et al., 2009; Su et al., 2012; Pereira et al., 2013).

Class Integron 1 can be considered as a marker for antibiotic resistance even without the presence of the gene cassette as they are usually located on the transposons or plasmids that carry other resistance genes (Leverstein-van Hall et al., 2003; Mokracka et al., 2011). This might explain why 90% of the integron class 1 positive isolates were found as multi-drug resistance (Tables 1, 2).

Among the 31 integron positive *E. coli* isolates, only seven (22%) were detected carrying gene cassettes. All the gene cassettes were detected in class 1 integron positive *E. coli* isolates. Five different gene cassettes were found in this study (Table 2), and these have been previously reported among clinical *E. coli* (Lim et al., 2009; Ho et al., 2012; Su et al., 2012; Koczura et al., 2013). It is interesting to note that the gene cassettes found in class 1 integron among *E. coli* isolates in Matang estuaries were also reported in *E. coli* isolates from swine (Lapierre et al., 2010), poultry (Soufi et al., 2009), and humans (Lim et al., 2009; Ho et al., 2012).

### Predominant phylogenetic groups in matang mangrove estuaries

The *E. coli* phylogenetic group B1 was the most prevalent (44%) in Matang mangrove estuaries, followed by phylogenetic groups A and D. Phylogenetic groups A and B1 comprised more than half (70%) of the total *E. coli* isolated from Matang mangrove estuaries. MLST genotyping has grouped phylogenetic groups A and B1 together, implying groups A and B1 to be sister groups (Gordon et al., 2008). Commensal *E. coli* was found to be predominantly in phylogenetic groups A and B1; whilst extraintestinal *E. coli* strains usually belong to groups B2 and D (Bingen et al., 1998; Picard et al., 1999; Johnson and Stell, 2000). Other studies have also reported that groups A and B1 were usually less pathogenic compared to groups B2 and D (Bingen et al., 1998; Picard et al., 1999; Johnson et al., 2001). Strains of A and B1 groups were recovered more frequently from aquatic environments than group B2 and D strains (Figueira et al., 2011). Walk et al. (2007) demonstrated that the majority of the *E. coli* strains that are able to persist in the environment belong to the B1 phylogenetic group. The findings from the present study are in agreement with previous studies in that A and B1 groups are predominant in the aquatic system, although phylogenetic group B1 comprised the most frequently isolated group in the present study. However, the distribution of these common phylogenetic groups did not hold true at station H, where phylogenetic group D was predomiant. Although, many studies have demonstrated groups B2 and D are usually more virulent (Bingen et al., 1998; Picard et al., 1999; Johnson et al., 2001), *E. coli* O157:H7 and other EHEC are in group D (Bidet et al., 2005), where cattle and birds (Escobar-Paìramo et al., 2006) are the main reservoirs. The findings obtained from the present study are realistic since station H was less influenced by human activity compared to the other stations where human activity was the main factor affecting the quality of the aquatic environment. Station H was least impacted by human activity where wild animals are thought to be the main source of *E. coli* at station H. However, due to the fact that pathogenic strains usually belong to group D, fecal contamination by feral animals can present a potential risk of pathogenic *E. coli*. Therefore, the identification of this kind of fecal contamination is necessary for appropriate management practices and remediation strategies to improve sustainable water resources.

Low prevalence of phylogenetic group B2 was found in our study which is in agreement with previous studies (Pereira et al., 2013). These isolates were detected in stations that were influenced more by human activities. It may show that this group is more associated with anthropogenic contamination as has been previously reported (Carlos et al., 2010). However, the percentage of *E. coli* in each group differs according to the sites of infection, geographical location, and level of antibiotic resistance (Duriez et al., 2001; Bukh et al., 2009). Carlos et al. (2010) reported that genotype B2_3_ was found only in human feces and could be a good indicator for human fecal contamination in water. In the present study, genotype B2_3_ was only detected from stations D, E, F, and G which were all located downstream of the fishing villages.

Many studies have reported the association of *E. coli* phylogenetic groups to their virulence, antibiotic resistance, and antibiotic resistant genes (Skurnik et al., 2005; Rijavec et al., 2006; Bukh et al., 2009; Garcia-Aljaro et al., 2009; Mataseje et al., 2009; Pereira et al., 2013; Mosquito et al., 2015). However, variable observations were reported from different studies. For instance, Mosquito et al. (2015) and Bukh et al. (2009) both reported that strains belonging to phylogenetic group D presented significantly higher percentages of multidrug resistance than the rest of the groups; while Skurnik et al. (2005), Rijavec et al. (2006), Garcia-Aljaro et al. (2009), Mataseje et al. (2009) and Pereira et al. (2013) had all found antibiotic resistance to be lower in *E. coli* phylogenetic group D and B2. In this study, we observed a lower percentage of antibiotic resistance in phylogenetic groups B2 (0%) and D (8%) as compared to group A (29%) and B1 (23%) (Table 1). However, the variation in observations from different studies could be due to the different origin of *E. coli.* Both studies (Bukh et al., 2009; Mosquito et al., 2015) reported an association of multidrug resistance with *E. coli* phylogenetic group D was of clinical origins but not environmental. Therefore, in our study, the higher percentage of multidrug resistance observed in group A and B1 was plausible. However, both *E. coli* phylogenetic group B2 and D demonstrated higher percentage of non-susceptibility to aminoglycosides, but not quinolones/fluoroquinolones. On the other hand, group A and B1 appear to be associated with resistance toward aminoglycosides and (fluoro) quinolones (Table 1 and Figure 3). The low non-susceptibility percentage in group D is out of our expectation as most studies have reported on its high resistance toward (fluoro) quinolones (Sabaté et al., 2008; Bukh et al., 2009; Mosquito et al., 2015). Probably, a study into the origins of *E. coli* phylogenetic group D isolated in this study will yield an explanation for this variation.

### Isolation of EAEC pathotype from matang mangrove estuaries

Our present finding also indicated that two isolates (1%) were pathogenic and identified as EAEC. Both EAEC isolates (SD4 and WGO3) belonged to phylogenetic group D and they were grouped in different clusters (Supplementary Table 4). Low percentage of the pathotypes in aquatic systems was also reported in a previous study (Hamelin et al., 2007). EAEC is known as an emergent pathogen causing diarrhea in developing countries and gastroenteritis outbreaks in industrialized countries, especially among children (Keskimäki et al., 2000; Villaseca et al., 2005). In Japan in 1993, a massive outbreak due to EAEC infection occurred among children (Keskimäki et al., 2000).

The source of EAEC remains unknown. The isolated EAEC were from stations D and G, and likely came from municipal wastewater as a potential source of pathogenic *E. coli*. The contaminated water may contaminate aquaculture products, while it is conceivable that their consumption could cause foodborne illness in humans. Water and food have been reported as the possible sources of EAEC infection in developing counties (Villaseca et al., 2005). This risk could become more significant if such bacteria are resistant to antibiotics.

Both EAEC isolates, SD4 and WGO3, contains class 1 integron with gene cassettes *aadA1* (1000 bp) and *dfrA5* (700 bp), respectively. The same gene cassettes with same length have been reported among clinical isolates of *E. coli* in the stools of infected persons in Malaysia (Lim et al., 2009; Ho et al., 2012). In addition, both EAEC isolates were MDR but were sensitive to fluoroquinolones. Fluoroquinolones has been found to be negatively associated with the presence of the virulence factors among the clinical *E. coli* strain (Johnson et al., 2003; Kawamura-Sato et al., 2010), but has not been observed among isolates from animals (Johnson et al., 2003; Kawamura-Sato et al., 2010). This may indicate that *E. coli* population in Matang estuaries is contributed by different sources of contamination.

### Genetic diversity using REP-PCR of *E. coli* isolates from matang mangrove estuaries

REP-PCR is known as a fast and discriminatory technique to characterize the genomic diversity of *E. coli* isolates (Rademaker et al., 2000). Although the present results indicate a high diversity of *E. coli* isolates, the cluster analysis showed that the 148 isolates could be grouped into seven clusters at 80% similarity.

Overall, 76 (51%) and 27 (18%) *E. coli* isolates from eight different sampling sites were grouped into cluster I and V, respectively. The close similarity between some of the *E. coli* isolates over large spatial scale reflects both the hydrodynamics and interconnected waterways that characterize the Matang estuarine system. However, the diverse and variable nature of the *E. coli* population could be due to their survival rate, growth and inputs from different sources in the aquatic environment and, which differ according to the strain characteristic (Anderson et al., 2006; Kon et al., 2007, 2009). This explains why eight *E. coli* isolates in station F were grouped into four different clusters in the present study, while diversity was less in station C (*n* = 8) with two clusters, and station D (*n* = 24) with three clusters.

Although Selinsing River (stations G and H) was less used for aquaculture production compared to Sangga Besar River, the diversity of *E. coli* isolates was slightly higher. Besides the prolonged persistence and adaptation of *E. coli* in the water, heterogeneity among isolates could be due to erosion of the river banks and the presence of more wildlife that act as vectors of *E. coli* transmission (Solo-Gabriele et al., 2000; Kon et al., 2009). The high diversity was also found at stations A, B, and E as these stations were located next to human settlements and fish cage farms (E). However, there is no a single source that contributes to *E.coli* diversity and multiple sources are more likely (Kon et al., 2009).

Several studies reported different sources of *E. coli* isolates such as bird droppings (Edge and Hill, 2005), wildlife (Somarelli et al., 2007), and humans (Ram et al., 2004). However, it was not the main aim of this study to trace the sources of *E. coli* in the estuarine system. Perhaps, future work should compare the REP-PCR of *E. coli* isolates with those from humans and known animals in order to determine the sources of *E. coli* isolates in the Matang area.

## Conclusion

Our study shows a high prevalence of the MDR (34%) among *E. coli* isolated from Matang estuaries. Phylogenetic group B1 has high resistance to antibiotics, thus playing a major role in the transmission of antimicrobial resistance via the aquatic environment. In particular, a high level of antibiotic resistance was observed at the upstream of the estuaries, which could be attributable to the adjacent human settlement and fish farms. Resistance to aminoglycosides, beta-lactams and tetracycline were most frequently detected in this study. Presence of integrons (21%) is another interesting result reflecting the contaminated MMFR estuaries that could act as a reservoir or source of MDR *E. coli* for the transmission of antibiotic resistance to the environment.

### Conflict of interest statement

The authors declare that the research was conducted in the absence of any commercial or financial relationships that could be construed as a potential conflict of interest.
