# Supplementary material for: Diverse and abundant multi-drug resistant E. coli in Matang mangrove estuaries, Malaysia
Source: Front Microbiol. 2015 Sep 29;6:977. doi: 10.3389/fmicb.2015.00977 (PMC4586456; doi:10.3389/fmicb.2015.00977)
Supplement: Supplementary file 1 [file Table1.PDF]

Supplementary Table 1. List of primer sequences

Purpose	Primer	Target	Oligonucleotide sequence (5' to 3')	Size (bp)	Reference
E. coli detection					
	PhoA-F	*PhoA*	GTCACAAAAGCCCGGACACCATAAATGCCT	903	Yu and Thong, 2009
	PhoA-R		TACACTGTCATTACGTTGCGGATTTGGCGT		
Genotyping					
	REP	REP	GCG CCG ICA TGC GGC ATT		Lim et al., 2009
Phylogrouping					
	ChuA.1	*chuA*	GACGAACCAACGGTCAGGAT	279	Clermont et al., 2000
	ChuA.2		TGCCGCCAGTACCAAAGACA		
	YjaA.1	*yjaA*	TGAAGTGTCAGGAGACGCTG	211	Clermont et al., 2000
	YjaA.2		ATGGAGAATGCGTTCCTCAAC		
	TspE4C2.1	TSPE4.C2	GAGTAATGTCGGGGCATTCA	152	Clermont et al., 2000
	TspE4C2.2		CGCGCCAACAAAGTATTACG		
Pathotyping					
	VT-F	VT	GAGCGAAATAATTTATATGTG	518	
	VT-R		TGATGATGGCAATTCAGTAT		Aranda et al., 2007
	eae-F	*eae*	CTGAACGGCGATTACGCGAA	917	
	eae-R		5′-CGAGACGATACGATCCAG		Aranda et al., 2007
	bfpA-F	*bfpA*	AATGGTGCTTGCGCTTGCTGC	326	
	bfpA-R		GCCGCTTTATCCAACCTGGTA		Aranda et al., 2007
	aggR-F	*aggR*	GTATACACAAAAGAAGGAAGC	254	
	aggR-R		ACAGAATCGTCAGCATCAGC		Aranda et al., 2007
	LT-F	LT	GCACACGGAGCTCCTCAGTC	218	Vidal et al., 2005
	LT-R		TCCTTCATCCTTTCAATGGCTTT		
	ST-F	ST	GCTAAACCAGTAGAG(C)TCTTCAAAA	147	Vidal et al., 2005
	ST-R		CCCGGTACAG(A)GCAGGATTACAACA		
	daaE-F	*daaE*	GAACGTTGGTTAATGTGGGGTAA	542	Vidal et al., 2005
	daaE-R		TATTCACCGGTCGGTTATCAGT		
	virF-F	*virF*	AGCTCAGGCAATGAAACTTTGAC	618	Vidal et al., 2005
	virF-R		TGGGCTTGATATTCCGATAAGTC		
	ipaH-F	*ipaH*	CTCGGCACGTTTTAATAGTCTGG	933	Vidal et al., 2005
	ipaH-R		GTGGAGAGCTGAAGTTTCTCTGC		
Integrons detection					
	IntI1-F	*IntI1*	GGTCAAGGATCTGGATTTGG	500	Machado et al., 2005
	IntI1-R		ACATGCGTGTAAATCATCGTC		
	IntI2-F	*IntI2*	CACGGATATGCGACAAAAAGGT	740	Machado et al., 2005
	IntI2-R		GTAGCAAACGAGTGACGAAATG		
	IntI3-F	*IntI3*	AGTGGGTGGCGAATGAGTG	600	Machado et al., 2005
	IntI3-R		TGT TCT TGT ATC GGC AGG TG		
	5CS	5'CS	GGCATCCAAGCAGCAAG		Machado et al., 2005
	3CS	3'CS	AAGCAGACTTGACCTGA		
	attI2-F	*attI2*	GACGGCATGCACGATTTGTA	2000	Machado et al., 2005
	orfX-R	*orfX*	GATGCCATCGCAAGTACGAG		
