# Supplementary material for: Diverse and abundant multi-drug resistant E. coli in Matang mangrove estuaries, Malaysia
Source: Front Microbiol. 2015 Sep 29;6:977. doi: 10.3389/fmicb.2015.00977 (PMC4586456; doi:10.3389/fmicb.2015.00977)
Supplement: Supplementary file 2 [file Table2.PDF]

Supplementary Table 2. Antibiotic susceptibility pattern of 148 isolates of E. coli isolated from Matang Mangrove Estuaries, Perak, Malaysia.

		Total # isolates	Aminoglycosides												Beta-lactams												Quinolones/ Fluoroquinolones												Tetracyclines			Phenicols			Sulpha group		
			N			S			K			CN			AMC			AMP			CRO			EFT			ENR			CIP			OA			NA			TE			C			SXT		
			S	I	R	S	I	R	S	I	R	S	I	R	S	I	R	S	I	R	S	I	R	S	I	R	S	I	R	S	I	R	S	I	R	S	I	R	S	I	R	S	I	R	S	I	R
station	A	17	3	12	2	10	2	5	14	2	1	15	1	1	16	0	1	14	0	3	16	0	1	16	0	1	14	2	1	16	0	1	15	0	2	14	1	2	11	0	6	15	0	2	16	0	1
	B	28	4	17	7	16	3	9	19	1	8	27	0	1	24	4	0	12	3	13	28	0	0	28	0	0	17	4	7	21	2	5	14	0	14	14	2	12	15	0	13	18	0	10	15	0	13
	C	8	3	4	1	6	0	2	7	0	1	8	0	0	8	0	0	6	1	1	8	0	0	8	0	0	5	3	0	7	1	0	5	0	3	5	3	0	5	0	3	7	0	1	8	0	0
	D	24	11	9	4	12	7	5	19	1	4	24	0	0	24	0	0	14	2	8	24	0	0	24	0	0	17	4	3	21	0	3	21	0	3	19	2	3	15	0	9	16	1	7	18	0	6
	E	20	4	15	1	14	2	4	18	2		20	0	0	20	0	0	16	3	1	20	0	0	20	0	0	19	1	0	20	0	0	20	0	0	19	1	0	19	0	1	19	1	0	20	0	0
	F	8	1	5	2	6	2		6	1	1	7	1	0	3	1	4	5	1	2	8	0	0	6	2	0	6	1	1	8	0	0	7	0	1	6	1	1	6	0	2	8	0	0	7	0	1
	G	19	5	12	2	17	0	2	15	1	3	19	0	0	13	1	5	11	3	5	18	0	1	18	0	1	16	2	1	18	0	1	16	0	3	15	2	2	13	0	6	17	0	2	15	0	4
	H	24	8	15	1	21	2	1	22	1	1	23	0	1	24	0	0	22	1	1	24	0	0	24	0	0	22	1	1	23	0	1	23	0	1	23	0	1	22	0	2	24	0	0	22	0	2
Phylogroup	A	38	9	23	6	28	4	6	30	2	6	36	0	2	33	0	5	22	5	11	37	0	1	37	0	1	25	8	5	33	2	3	27	0	11	27	3	8	26	0	12	30	1	7	29	0	9
	B1	65	15	38	12	44	7	14	49	3	13	64	1	0	59	3	3	43	7	15	65	0	0	64	1	0	48	9	8	57	1	7	50	0	15	45	8	12	44	0	21	52	1	12	51	0	14
	B2	7	4	3		2	3	2	7	0	0	7	0	0	5	1	1	6	0	1	7	0	0	7	0	0	7	0	0	7	0	0	7	0	0	7	0	0	5	0	2	7	0	0	6	0	1
	D	38	11	25	2	28	4	6	34	4	0	36	1	1	35	2	1	29	2	7	37	0	1	36	1	1	36	1	1	37	0	1	37	0	1	36	1	1	31	0	7	35	0	3	35	0	3
Season	Wet	56	14	39	3	54	1	1	50	3	3	56	0	0	56	0	0	42	9	5	56	0	0	56	0	0	53	0	3	53	0	3	52	0	4	51	1	4	51	0	5	51	2	3	53	0	3
	Dry	92	25	50	17	48	17	27	70	6	16	87	2	3	76	6	10	58	5	29	90	0	2	88	2	2	63	18	11	81	3	8	69	0	23	64	11	17	55	0	37	73	0	19	68	0	24
TOTAL		148	39	89	20	102	18	28	120	9	19	143	2	3	132	6	10	100	14	34	146	0	2	144	2	2	116	18	14	134	3	11	121	0	27	115	12	21	106	0	42	0	2	22	121	0	27

The figure is number of isolates

Antibiotic. N, neomycin; S, streptomycin; K, kanamycin; CN, gentamicin; AMC, amoxicillin/clavulanic acid; AMP, ampicillin; CRO, ceftriazone; EFT, ceftiofur; ENR, enrofloxacin; CIP, ciprofloxacin; OA, oxolinic acid; NA, nalidixic acid; TE, tetracycline; C, chloramphenicol; SXT, sulfamethoxazole/trimetroprim.

Antibiotic resistance. S, sensitive; I, Intermediate; R, resistant.
