# Supplementary material for: Diverse and abundant multi-drug resistant E. coli in Matang mangrove estuaries, Malaysia
Source: Front Microbiol. 2015 Sep 29;6:977. doi: 10.3389/fmicb.2015.00977 (PMC4586456; doi:10.3389/fmicb.2015.00977)
Supplement: Supplementary file 3 [file Table3.PDF]

Supplementary Table 3. Summary of results to determine the correlation (Rn) between the presence of integras genes (*IntI*1 and *IntI*1+*IntI*2) and antibiotic resistance in 148 isolates of *E. coli*. Chi-square value for significance test based on chi-square contingency test with correction for continuity.

**Antibiotics**	I				II				III				IV	V	VI
	N	S	K	CN	AMC	AMP	CRO	EFT	ENR	CIP	OA	NA	TE	C	SXT
IntI1															
Rn	-	**0.50**	**0.66**	0.65	0.58	**0.55**	0.64	**0.66**	**0.66**	**0.72**	**0.65**	**0.62**	**0.66**	**0.69**	**0.81**
Chi-square	0.03	17.54	26.05	3.50	3.13	28.51	0.06	5.40	30.39	25.52	22.33	23.50	42.68	27.74	64.52
p-value	0.85	<0.01	<0.01	0.06	0.07	<0.01	0.80	0.02	<0.01	<0.01	<0.01	<0.01	<0.01	<0.01	<0.01
IntI1+IntI2															
Rn	-	0.41	**0.65**	0.91	0.73	0.38	0.92	0.89	**0.62**	0.81	**0.66**	**0.61**	**0.49**	**0.73**	**0.69**
Chi-square	0.41	1.89	5.09	1.05	0.01	1.70	3.83	1.50	10.53	3.77	5.40	10.09	7.07	15.38	13.22
p-value	0.52	0.17	0.02	0.31	0.91	0.19	0.05	0.20	<0.01	0.05	0.02	0.02	<0.01	<0.01	<0.01
