# Supplementary material for: Diverse and abundant multi-drug resistant E. coli in Matang mangrove estuaries, Malaysia
Source: Front Microbiol. 2015 Sep 29;6:977. doi: 10.3389/fmicb.2015.00977 (PMC4586456; doi:10.3389/fmicb.2015.00977)

Supplementary Table 4 . Dendrogram of REP-PCR profiles using UPGMA based on Dice coefficients similarity; isolates are divided into seven clusters; I, II, III, IV, V, VI, and VII. Antibiotic resistance profile; (1: Aminoglycosidase; 2: Beta-lactams; 3: Fluroquinolone; 4: Quinolone; 5: Tetracycline; 6: Phenicols; 7: Sulfonamide; R-Ph: resistant phenotype. Antibiotic susceptibility pattern, (black) the isolate is not-susceptible to all agents listed in category, (gray) the isolate is non-susceptible to some, but not all agents listed in category, (white) the isolate is susceptible to all agents listed in category. The yellow highlighted isolates represent EAEC.

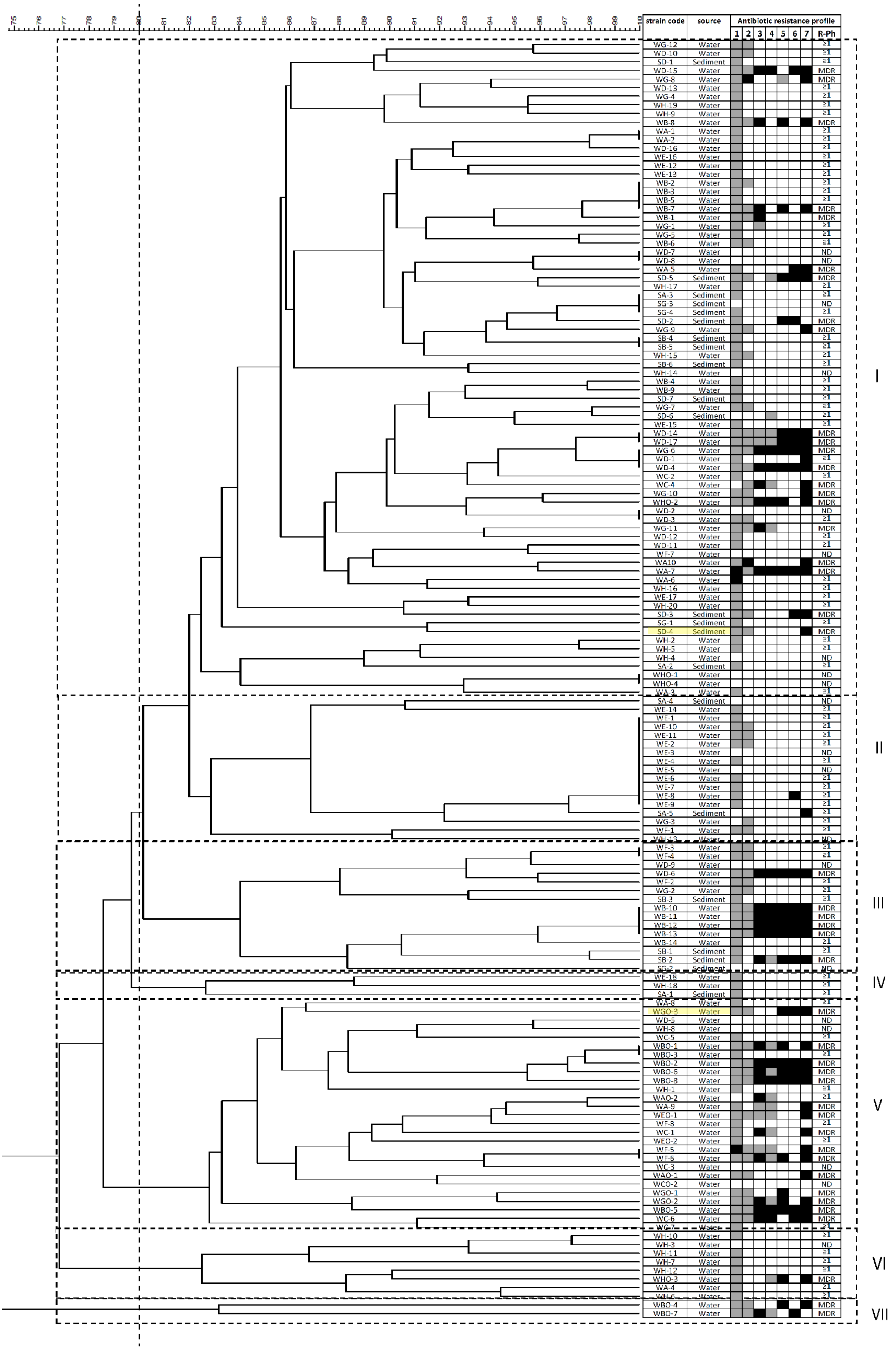

Supplement: Supplementary file 4 [file Table4.PDF]
